# Mapping the EORTC QLQ-C30 and QLQ-H&N35 to the EQ-5D for head and neck cancer: Can disease-specific utilities be obtained?

**DOI:** 10.1371/journal.pone.0226077

**Published:** 2019-12-13

**Authors:** Ann-Jean C. C. Beck, Jacobien M. Kieffer, Valesca P. Retèl, Lydia F. J. van Overveld, Robert P. Takes, Michiel W. M. van den Brekel, Wim H. van Harten, Martijn M. Stuiver

**Affiliations:** 1 Department of Head and Neck Oncology and Surgery, the Netherlands Cancer Institute, Amsterdam, the Netherlands; 2 Division of Psychosocial Research and Epidemiology, the Netherlands Cancer Institute, Amsterdam, the Netherlands; 3 Department of Health Technology and Services Research, University of Twente, Enschede, the Netherlands; 4 Radboud University Medical Center, Radboud Institute for Health Sciences, Scientific Center for Quality of Healthcare, Nijmegen, the Netherlands; 5 Department of Otolaryngology and Head and Neck surgery, Radboud University Medical Center, Radboud Institute for Health Sciences, Nijmegen, the Netherlands; 6 Institute of Phonetic Sciences, University of Amsterdam, Amsterdam, the Netherlands; 7 Department of Oral and Maxillofacial Surgery, Amsterdam University Medical Center, Amsterdam, the Netherlands; 8 Department of Clinical Epidemiology Biostatistics and Bioinformatics, Amsterdam University Medical Center, University of Amsterdam, Amsterdam, the Netherlands; University of Colorado Denver, UNITED STATES

## Abstract

**Introduction:**

Innovations in head and neck cancer (HNC) treatment are often subject to economic evaluation prior to their reimbursement and subsequent access for patients. Mapping functions facilitate economic evaluation of new treatments when the required utility data is absent, but quality of life data is available. The objective of this study is to develop a mapping function translating the EORTC QLQ-C30 to EQ-5D-derived utilities for HNC through regression modeling, and to explore the added value of disease-specific EORTC QLQ-H&N35 scales to the model.

**Methods:**

Data was obtained on patients with primary HNC treated with curative intent derived from two hospitals. Model development was conducted in two phases: 1. Predictor selection based on theory- and data-driven methods, resulting in three sets of potential predictors from the quality of life questionnaires; 2. Selection of the best out of four methods: ordinary-least squares, mixed-effects linear, Cox and beta regression, using the first set of predictors from EORTC QLQ-C30 scales with most correspondence to EQ-5D dimensions. Using a stepwise approach, we assessed added values of predictors in the other two sets. Model fit was assessed using Akaike and Bayesian Information Criterion (AIC and BIC) and model performance was evaluated by MAE, RMSE and limits of agreement (LOA).

**Results:**

The beta regression model showed best model fit, with global health status, physical-, role- and emotional functioning and pain scales as predictors. Adding HNC-specific scales did not improve the model. Model performance was reasonable; R^2^ = 0.39, MAE = 0.0949, RMSE = 0.1209, 95% LOA of -0.243 to 0.231 (bias -0.01), with an error correlation of 0.32. The estimated shrinkage factor was 0.90.

**Conclusions:**

Selected scales from the EORTC QLQ-C30 can be used to estimate utilities for HNC using beta regression. Including EORTC QLQ-H&N35 scales does not improve the mapping function. The mapping model may serve as a tool to enable cost-effectiveness analyses of innovative HNC treatments, for example for reimbursement issues. Further research should assess the robustness and generalizability of the function by validating the model in an external cohort of HNC patients.

## Introduction

Over the years, new treatment regimens, including innovative medical devices, have been emerging in the field of head and neck cancer (HNC) to improve quality of life of patients. In the process of securing access to these innovations for HNC patients, reimbursement plays a key role. Before reimbursement of clinical innovations is considered by governing bodies, an economic evaluation is often required.

This evaluation can be performed when data regarding costs of the treatment and quality of life of patients are available, provided that quality of life is expressed in quality-adjusted life years (QALYs). To calculate QALYs, utilities are necessary and these can be derived from preference-based measures (PBMs), such as the EuroQol five-dimensional questionnaire (EQ-5D) [1]. In clinical practice, however, utility data are not routinely collected by means of PBMs. The resulting unavailability of QALYs hinders the cost-effectiveness evaluation that is needed for clinical implementation of innovative treatments and to inform healthcare providers on the cost-effectiveness of existing treatment options.

While often not using PBMs, studies evaluating the effectiveness of head and neck cancer (HNC) treatments or devices frequently do use health-related quality of life (HRQoL) instruments, such as the European Organization for Research and Treatment of Cancer Quality of Life Questionnaire-Core 30 (EORTC QLQ-C30) and the disease-specific EORTC QLQ module for HNC (QLQ-H&N35) [2, 3]. Such (disease-specific) HRQoL measures could be used to estimate utilities, by making use of a ‘mapping model’[4, 5]. For this purpose, several types of regression models can be employed, each with their own advantages and disadvantages [6].

The most commonly used regression method in the mapping literature is the ordinary least squares (OLS) linear regression [7, 8]. The linear regression is a simplistic model, which is easily applicable in practice. However, the model may over- or undershoot the utility interval [0 to 1]. Also, assumptions related to the regression are often violated, e.g. homoscedasticity and normal distribution of residuals. To deal with the often skewed distribution and ceiling effects of HRQoL scores, the Tobit model has been suggested as an alternative model for mapping functions [9, 10]. This model however, assumes an underlying (but unobserved) normal distribution of the data. The comparable semiparametric Cox proportional hazards model shares the advantages of the Tobit model for dealing with non-normal data, but without the undesirable parametric assumption [11]. Even so, it is less straightforward to interpret and has not been frequently used for mapping purposes. Finally, beta regression uses a beta distribution, which can shape according to the skewness of the data often seen in PBM data. The regression model accommodates a dependent variable that is limited to an interval of 0 to 1, but cannot handle the extreme values (0 and 1) on the boundaries of this interval [6, 12]. Currently, there is no consensus on which statistical method to use in the development of mapping models.

Previous studies have concluded that estimating EQ-5D utilities using outcomes of the EORTC QLQ-C30 is feasible for several forms of cancer [4, 5, 7, 8, 13]. To the best of our knowledge, no mapping model based on the EORTC QLQ-C30 has been developed to date for use in a HNC population. Therefore, the primary objective of the current study was to develop an optimal mapping model to estimate utilities, required for economic evaluation, by translating the generic EORTC QLQ-C30 outcomes to EQ-5D utilities for HNC patients, comparing different statistical approaches.

HNC-specific symptoms have a substantial influence on the HRQoL of patients, but are not all addressed in the EORTC QLQ-C30. Adding (parts of) the EORTC QLQ-HN35 module to the mapping model might further improve utility estimations, but current evidence about the value of using cancer type-specific QLQ scales for mapping models is scarce. [4, 5] Hence, the secondary objective of the study was to explore the added value of the EORTC QLQ-H&N35 scales to the mapping model.

Ultimately, the aim of this study is to facilitate economic evaluation of health care innovations for patients with HNC, in case of absence of utility data, and thereby support the implementation of such innovations in clinical practice.

## Material and methods

### Data source

Data used for the purpose of the current study were collected for the Dutch Head and Neck Audit (DHNA). The purpose of the audit is to measure and monitor the quality of care in the Dutch HNC centers using a set of quality indicators. The quality indicators have been described in detail elsewhere [14]. This audit collects data prospectively by means of an online survey completed by HNC patients. Patients diagnosed with primary HNC (head and neck squamous cell carcinoma (HNSCC) and salivary gland malignancies) and treated with curative intent are included in the audit. Exclusion criteria for the DHNA are: patients with other types of head and neck malignancies (e.g. skin cancer, sarcomas and esthesioneuroblastoma), a second primary tumor or with recurrent disease.

In the DHNA, quality of life is measured routinely using the EORTC QLQ-C30, EORTC QLQ-H&N35 and three-level EQ-5D (EQ-5D-3L) at diagnosis (baseline) and 3, 6 12 and 24 months after end of treatment. The register also incorporates patient and clinical characteristics, including age, sex, tumor site, treatment and TNM stage. For the current study, datasets were available from patients treated in the Netherlands Cancer Institute (NKI) and Radboud University Medical Center (Radboudumc) between November 2014 and February 2017. Only cases with complete quality of life data (i.e. no missing scale scores on the QOL questionnaires) were included in the study. The data was de-identified to ensure the anonymity of the patients. The procedures were in accordance with the ethical standards of the ethics committee of the Radboudumc (registration number: 2014/070). Approval for this study was obtained by the ethics committee of the Netherlands Cancer Institute (NKI) (registration number: METC16.0502). The participating hospitals are: the NKI, Amsterdam and Radboudumc, Nijmegen. A written informed consent was obtained from all participants upon participation.

### Instruments

The EORTC QLQ-C30 is a generic instrument that is developed to assess HRQoL in cancer patients. The EORTC QLQ-C30 consists of 30 questions, resulting in a two-item global health status/QoL scale, five multi-item functional scales (physical functioning, role functioning, emotional functioning, cognitive functioning and social functioning), three multi-item symptom scales (fatigue, nausea/vomiting and pain) and six single-item symptom scales (dyspnea, insomnia, appetite loss, constipation, diarrhea and financial difficulties) [2].

The HNC-specific EORTC QLQ-H&N35 module is a supplement, containing seven multi-item scales (pain, swallowing, senses problems, speech problems, trouble with social eating and social contact, and less sexuality) and eleven single-item scales (teeth, opening mouth, dry mouth, sticky saliva, coughing, felt ill, pain killers, nutritional supplements, feeding tube, weight loss, weight gain) [3]. Both the EORTC QLQ-C30 and QLQ-H&N35 scales employ a 4-point response format (‘‘not at all” to ‘‘very much”), with the exception of the global QoL scale, which has a 7-point response format. Scale scores are transformed to a scale from 0 to 100 according to the EORTC scoring algorithm [15]. For the functioning and the global QoL scale, a higher score indicates better health. For the symptoms scales, a higher score indicates a higher level of symptom burden [3, 15].

The EQ-5D-3L is a generic preference-based instrument that functions as a health state classifier and consist of five dimensions: mobility, self-care, usual activities, pain/discomfort and anxiety/depression. The EQ-5D has three levels of functioning: no problems, some problems, and extreme problems. It provides 243 health profiles in total, which are often reported as vectors ranging from 11111 (full health) to 33333 (worst health). Health profiles can be converted into a utility index score by applying the EQ-5D scoring algorithm [1]. The utility index score, a number from 0 to 1, reflects the HRQoL, and is used for cost-effectiveness purposes. Utility values are adjusted for the respective country by means of a tariff applied in the mapping methods. In this study, the Dutch tariff was used.

### Model development

**Fig 1:**
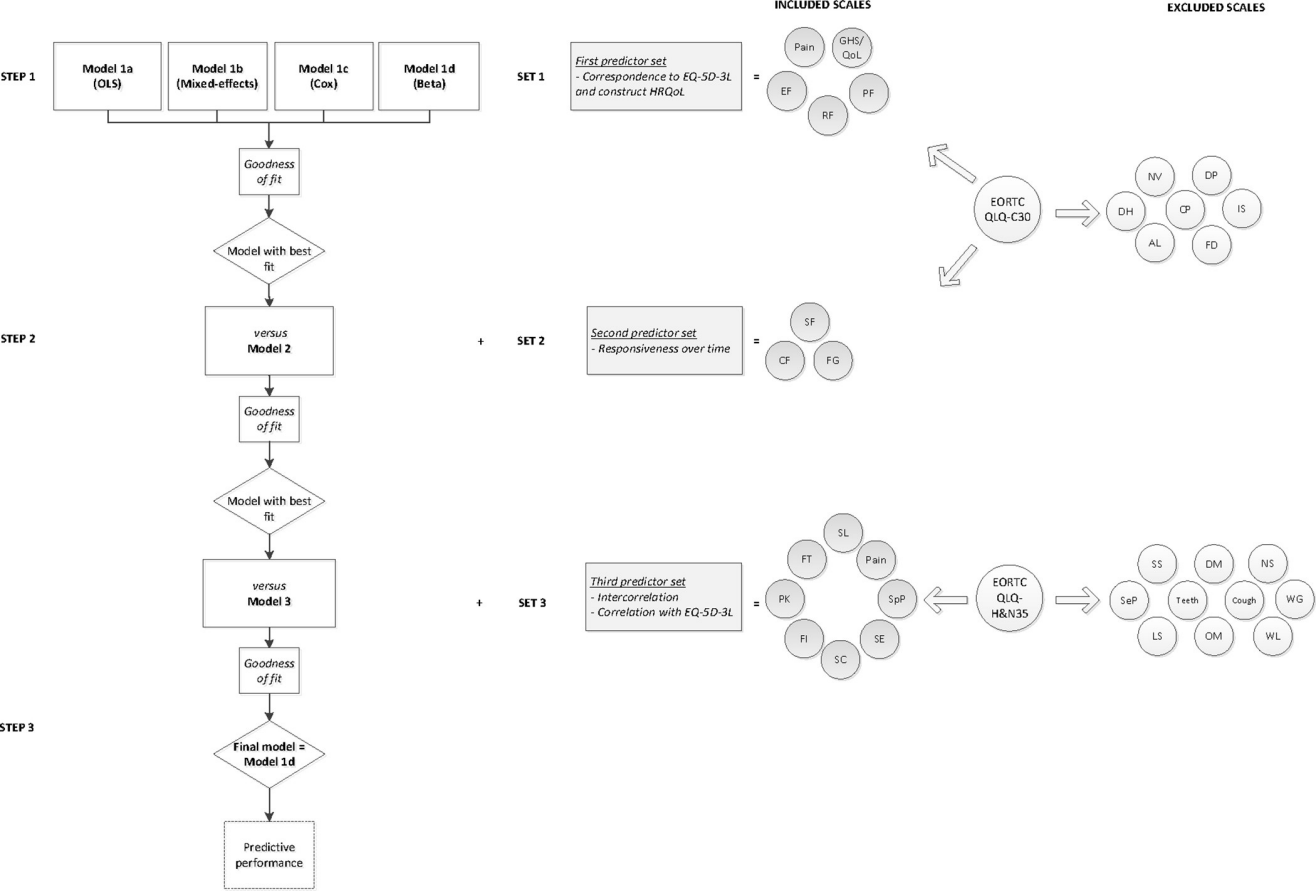
Flowchart of model development. The grey rectangles display the three predictor sets with their criteria; the white rectangles the different models. The squares indicate the assessment of the models during model comparison; the rhombuses indicate the decision-making in the process. Assessment of the model performance is displayed with a dotted line. The EORTC QLQ-C30 and QLQ-H&N35 scales included in the predictor sets are highlighted in grey circles. The scales that were excluded are colored white. Abbreviations: AL, appetite loss; Cough, coughing; CF, cognitive functioning; CP, constipation; DH, diarrhea; DP, dyspnea; DM, dry mouth; EF, emotional functioning; EORTC, European Organization for Research and Treatment of Cancer; EQ-5D-3L, three-level EuroQol five-dimensional questionnaire; FD, financial difficulties; FG, fatigue; FI, felt ill; FT, feeding tube; GHS/QoL, global health status/quality of life; HRQoL, health-related quality of life; IS, insomnia; LS, less sexuality; NT, nutritional supplements; NV, nausea and vomiting; OLS, ordinary least-squares, OM, opening mouth; PF, physical functioning; PK, pain killers; Quality of Life Questionnaire-Core 30; QLQ-H&N35, Quality of Life Questionnaire-Head and Neck35; RF, role functioning; SC, trouble with social contact; SE, trouble with social eating; SpP, speech problems; SeP, senses problems; SL, swallowing; SF, social functioning; SS, sticky saliva; WG, weight gain; WL, weight loss.

Development of the best fitting mapping model was conducted in two phases. In the first phase, QLQ scales were preselected as potential predictors, resulting in three predictor sets based on theory (Set 1) and combined theory- and data-driven considerations (Set 2 and 3). This was done to retain parsimony of the model. In the second phase, statistical analyses were performed in three consecutive steps in order to select a model with the best fit, considering the different predictor sets. A schematic overview is given in Fig 1.

In this Methods section, both phases are described separately.

#### Preselection of QLQ scales

Three sets of HRQoL outcomes were selected as potential predictors to map onto the EQ-5D. The first set of predictors was selected from EORTC QLQ-C30 scales based on the correspondence of these scales with the EQ-5D dimensions and its underlying construct. Correspondence was evaluated by matching EORTC QLQ-C30 scales to EQ-5D dimensions based on degree of overlap in content between items in both questionnaires. This predictor set functioned as a base for the model, and was retained in the model throughout the predictor selection from Set 2 and Set 3.

The second set of predictors included a number of the remaining EORTC QLQ-C30 scales, which were selected based on their ability to reflect on changes over time (a.k.a. responsiveness). A literature search was conducted to estimate the responsiveness of the scales. Studies were considered eligible when HNC patients had undergone a surgical and/or organ sparing intervention, the EORTC QLQ-C30 was completed at least twice by these patients at various time points within a timeframe of at least three months in which responsiveness of QoL was expected based on the treatment, and the sample size was ≥100. The search was restricted to studies published between January 2012 and July 2017. From the included studies, effect sizes (ES) were calculated for each EORTC QLQ-C30 scale, by dividing the mean difference of the score by the pooled standard deviation, and compared with the average ES of the EQ-5D calculated with the data used in this study [16].

A third set containing individual predictors consisting of EORTC QLQ-H&N35 scales was developed to explore whether use of HNC-specific HRQoL outcomes could improve the fit of the mapping model. Scales were assessed on intercorrelation, to limit overfitting as well as prevent multicollinearity. If a Pearson correlation coefficient of ≥ 0.7 between two individual EORTC QLQ-H&N35 scales was present, one of the scales was excluded based on theoretical considerations. Of the remaining EORTC QLQ-H&N35 scales, those that correlated with the dependent outcome (Pearson correlation coefficient ≥ 0.3) were included in the third set of predictors. The data showed no outliers, but were not entirely normally distributed. For completeness, we re-ran the analyses on the basis of Spearman's correlation results.

The predictors were tested one by one for their additional value to the model.

#### Statistical analysis

The statistical analysis was conducted in three steps (Fig 1). The first step consisted of selecting the best fitting regression method using only the first set of predictors as input for the models. We considered four commonly used regression models:

1. Regression analysis using an OLS estimator (Model 1a);
2. Mixed-effects modeling approach (Model 1b) using a maximum likelihood solution, with a random intercept to take into account mutual correlation within repeated measurements present in our data;
3. Cox regression (Model 1c) with ‘censoring’ of all EQ-5D utility index scores <1.
4. Classical beta regression (Model 1d) modeling the dependent variable y in a unit interval 0 < y < 1. In order to include the full health (utility value of 1) in this interval, a transformation of y was applied [17, 18]:

(y·(n−1)+0.5)/n

in which y is the utility and n is the sample size. To create a generalized linear model, we applied the logit link function [17].

To select the overall best statistical approach, we compared the four models using the Akaike Information Criterion (AIC) and Bayesian Information Criterion (BIC) [19, 20]. The AIC and BIC can be used to compare non-nested models and reflect the relative quality of the models by assessing the goodness of fit while penalizing the number of model parameters. Models with lower BIC or AIC values are considered to be better fitting models, although there is much debate about how to interpret the numerical differences in outcomes between models. Published rules of thumb are: a between model difference in the AIC or BIC of 0 to 2 is considered to be weak, 2 to 6 to be positive, 6 to 10 to be strong and above 10 to be very strong [20, 21]. In this study, we considered the regression method with the lowest AIC and BIC to be the most appropriate base model to use for the subsequent statistical steps.

In the second step, we extended the base model selected in step 1 with the second set of predictors containing all responsive EORTC QLQ-C30 scales (Model 2). The added value of these predictors was assessed using the AIC, BIC and likelihood-ratio (LR) test with a cutoff p-value of 0.05. In case of significant outcome (p<0.05) of the LR test and lower AIC and BIC values, we used manual stepwise backward elimination of predictors of the second set, for parsimony of the model. Backward elimination was based on the p-value of the coefficients (using a cutoff of 0.1).

In step 3, we explored the added value of the selected EORTC QLQ-H&N35 predictors (third set) for each variable separately (Model 3). Each of these models was compared to the model with the best fit so far obtained after step 2. The same model fit statistics were used as described in step 2.

### Assessment of predictive performance and validation of final model

Predictive performance of the final model was evaluated with the R squared (R^2^), the mean absolute error (MAE) and root mean square error (RMSE). In addition, the 95% limits of agreement (LOA) of observed and predicted EQ-5D utilities and the correlation coefficient of the error were determined by means of a Bland-Altman analysis [13, 22].

The estimated shrinkage factor (*s*) of the coefficients of the final model was calculated to adjust for inflated regression coefficients, and thus improve generalizability of the model to the target population. A heuristic formula was used:

s=(modelχ2−df)/modelχ2

in which model χ2 indicates the likelihood ratio χ2 of the model, and df stands for the degrees of freedom of the candidate predictors involved in the model [23].

We cross-validated the model fit by calculating MAE and RMSE of models fitted in 1000 bootstrap samples (with replacement) as applied to the original data as a means to assess the robustness of the predictive performance and internal validation.

Statistical analyses were performed in R version 3.4.3. (2017-11-30).

## Results

### Descriptive analyses

Details on patient, tumor and treatment characteristics, including the response rate to the EORTC QLQ-C30 and QLQ-H&N35, are listed in Table 1.

**Table 1:** Patient, tumor and treatment characteristics. Tumors were staged according to cTNM clinical classification of the Union for International Cancer Control (UICC) (2009, 7^th^ edition) [24].

Characteristics	Total no. (%)
Mean age, y (range)	63.0 (30.3–90.6)
Median age, y (range)	62.4 (30.3–90.6)
Sex	
Male	172 (72.9)
Female	64 (27.1)
Smoking	
Never	49 (20.8)
Stopped	106 (44.9)
Current	77 (32.6)
Missing	4 (1.7)
Alcohol	
Never	60 (25.4)
Stopped	21 (8.9)
Current	153 (64.8)
Missing	2 (0.8)
Subsite	
Hypopharynx	18 (7.6)
Larynx	46 (19.5)
Nasopharynx	8 (3.4)
Oral cavity	55 (23.3)
Oropharynx	64 (27.1)
Sinonasal malignancies	15 (6.4)
Salivary glands	16 (6.8)
Unknown primary	14 (5.9)
cT classification	
T0	14 (5.9)
Tis	5 (2.1)
T1	69 (29.2)
T2	79 (33.5)
T3	35 (14.8)
T4	34 (14.3)
cN classification	
N0	121 (51.3)
N1	32 (13.6)
N2	81 (34.4)
N3	2 (0.8)
cM classification	
Mx	15 (6.4)
M0	221 (93.6)
Treatment	
Surgery	48 (20.3)
Surgery + RT	42 (17.8)
Surgery + CRT	5 (2.1)
RT	79 (33.5)
CRT	54 (22.9)
BRT	8 (3.4)
Response rate*	
Baseline	117 (32.4)
3 months FU	84 (23.3)
6 months FU	91 (25.2
12 months FU	49 (13.6)
24 months FU	20 (5.5)
Total	361 (100)
Completed questionnaires per patient	
1 questionnaire	236 (100)
2 questionnaires	89 (38)
3 questionnaires	32 (14)
4 questionnaires	4 (2)
5 (all) questionnaires	0 (0)

*Time since diagnosis can be calculated 7 to 9 weeks prior to end of treatment.

Abbreviations: BRT, bioradiation; CRT, chemoradiotherapy; FU, follow-up; RT, radiotherapy.

In total, 361 measurements of 236 patients were included in this study. Only complete cases were included in the study. Of the 236 patients, 73% were male with a mean age of 63 years. The majority of carcinomas were situated in the oropharynx (27%), oral cavity (23%) and larynx (19%). Almost 30 percent of the patients had an advanced (T3 or T4) tumor. Most common treatment modalities were surgery (20%), surgery with postoperative RT (17%), RT alone (34%) and chemoradiation (CRT, 23%). The response rate to the questionnaires in the data used in this study varied from 6% to 32% at the different time points.

The mean utility value of the study population was estimated at 0.83 (range 0.11–1.00; SD 0.18) (Table 2). In total, 33 unique health states were observed. Optimal health (utility = 1) was observed 123 times (34%). Worst HRQoL scores were found on the EORTC QLQ-C30 global health status/QoL (functional) scale (mean 73.87; SD 18.42) and fatigue (symptom) scale (mean 26.72; SD 22.87), and of the EORTC QLQ-H&N35 scales on the pain killers scale (38.50; SD 48.73).

**Table 2:** Summary results of HRQoL data derived from 361 observations.

EORTC QLQ-C30 scores	Mean (range)	SD
***Functional scales***		
Global health status/QoL	73.87 (16.67–100.00)	18.42
Physical functioning	87.37 (26.67–100.00)	16.27
Role functioning	79.13 (0.00–100.00)	24.53
Emotional functioning	80.06 (0.00–100.00)	20.86
Cognitive functioning	87.35 (33.33–100.00)	16.99
Social functioning	84.11 (0.00–100.00)	21.05
***Symptom scales***		
Fatigue	26.72 (0.00–100.00)	22.87
Nausea and vomiting	4.76 (0.00–100.00)	13.31
Pain	20.18 (0.00–100.00)	24.75
Dyspnea	10.25 (0.00–100.00)	20.10
Insomnia	23.00 (0.00–100.00)	27.06
Appetite loss	17.17 (0.00–100.00)	26.77
Constipation	8.77 (0.00–100.00)	19.72
Diarrhea	6.37 (0.00–100.00)	16.27
Financial difficulties	11.08 (0.00–100.00)	22.64
EORTC **QLQ-H&N35 scores**		
***Symptom scales***		
Pain	22.32 (0.00–100.00)	24.35
Swallowing	17.04 (0.00–100.00)	21.98
Senses problems	16.02 (0.00–100.00)	21.36
Speech problems	18.25 (0.00–100.00)	22.32
Trouble with social eating	19.34 (0.00–100.00)	20.66
Trouble with social contact	7.09 (0.00–100.00)	13.41
Less sexuality	21.56 (0.00–100.00)	30.13
Teeth	14.50 (0.00–100.00)	27.26
Opening mouth	6.65 (0.00–100.00)	19.53
Dry mouth	11.73 (0.00–100.00)	23.85
Sticky saliva	21.79 (0.00–100.00)	30.52
Coughing	20.31 (0.00–100.00)	26.17
Felt ill	5.36 (0.00–00.00)	15.78
Pain killers	38.50 (0.00–100.00)	48.73
Nutritional supplements	21.88 (0.00–100.00)	41.40
Feeding tube	4.16 (0.00–100.00)	19.98
Weight loss	24.93 (0.00–100.00)	43.32
Weight gain	19.67 (0.00–100.00)	39.80
**EQ-5D-3L score**		
Utility value	0.83 (0.11–1.00)	0.18

Abbreviations: EORTC, European Organization for Research and Treatment of Cancer; EQ-5D-3L, three-level EuroQol five-dimensional questionnaire; QLQ-C30, Quality of Life Questionnaire-Core 30; QLQ-H&N35, Quality of Life Questionnaire-Head and Neck35; SD, standard deviation.

### Model development

#### Preselected QLQ scales

For the first set of predictors, we selected physical functioning, role functioning, emotional functioning and pain, as these scales corresponded best to the EQ-5D dimensions mobility, daily activities, anxiety/depression and pain/discomfort respectively. No overlapping item was found for the self-care dimension of the EQ-5D. Global health status/QoL scale was also included in the first predictor set to reflect the broader construct of HRQoL. These five EORTC QLQ-C30 scales were considered the basic scales of the mapping model.

Out of the five eligible articles derived from the literature search, the social functioning, cognitive functioning and fatigue scales were found sufficiently responsive as the ES was ≥ 0.3, corresponding to the calculated average ES of the EQ-5D in this study. Consequently, these EORTC QLQ-C30 scales selected for the second set of predictors [25–29].

For the third set, the opening mouth, dry mouth and sticky saliva scales were excluded because they had a correlation of ≥ 0.7 with the social eating scale. This correlation was also observed between the scales felt ill and social contact. However, as these scales clearly cover different clinical aspects, both scales were considered for the third set. Eventually, eight EORTC QLQ-H&N35 scales were included in the third set of predictors based on their correlation (Pearson correlation coefficient ≥ 0.3) with the EQ-5D outcome: pain, swallowing, speech problems, trouble with social eating and social contact, felt ill, pain killers and feeding tube. We found similar results based on Spearman correlations, except for a lower correlation between the “feeding tube dependency” scale and the EQ-5D (r < 0.3), and less indications for collinearity between subscales "opening mouth" and “dry mouth " with “trouble with social eating" and “felt ill” with “trouble with social contact”. Re-running the analyses based on these correlations did not change the final results and conclusions.

#### Selection of statistical method

Based on the AIC and BIC, the beta regression method (Model 1d) showed the best relative goodness of fit and was therefore considered as the base model including the first predictor set for subsequent steps.

Supplementation of the base model with the second predictor set (Model 2) did not provide a better fit. In addition, the LR test was not significant (p = 0.55). Therefore, after step 2, the base model (Model 1d) was retained as the model with best fit.

In the third step, Models 3a to 3h were generated by adding HNC-specific scales individually to the base model. The addition of HNC-specific scales pain (Model 3b), pain killers (Model 3g) and feeding tube (Model 3h) resulted in a lower AIC compared to Model 1d. The BIC of these models however, were higher than the BIC of Model 1d. The LR test was significant only for Model 3b and Model 3g (p = 0.05 and p = 0.02 respectively). Based on parsimony and because of the ambiguity of the above results—none of the models including HNC-specific scales satisfied all three criteria for improved model fit—Model 1d was considered as the final model. Details on model fit statistics for all models are reported in Table 3. The parameter estimates of the final model are listed in Table 4.

**Table 3:** Summary results of the regression models.

	AIC	BIC	RMSE	MAE	LR test p-value
**Model 1a (OLS)**	-486.04	-458.81	0.1211	0.0915	
**Model 1b (mixed-effects model)**	-486.64	-455.53	0.1042	0.0784	
**Model 1c (Cox regression)**	2381.50	2400.94	1.5225	1.3241	
**Model 1d (beta regression)**	-1029.93	-1002.71	0.1209	0.0949	
**Model 2**	-1026.04	-987.15	0.1209	0.0952	0.55
Model 3*					
**Model 3a**	-1029.43	-998.321	0.1211	0.0949	0.22
**Model 3b**	-1031.78	-1000.67	0.1214	0.0955	0.05
**Model 3c**	-1027.93	-996.82	0.1209	0.0949	1.00
**Model 3d**	-1028.03	-996.91	0.1209	0.0948	0.76
**Model 3e**	-1028.55	-997.44	0.1207	0.0946	0.43
**Model 3f**	-1027.97	-996.86	0.1209	0.0949	0.84
**Model 3g**	-1033.60	-1002.49	0.1208	0.0945	0.02
**Model 3h**	-1030.88	-999.77	0.1196	0.0939	0.09

*Model 1d supplemented with eight EORTC QLQ-H&N35 scales individually: swallowing (3a), pain (3b), speech problems (3c), social eating (3d), social contact (3e), felt ill (3f), pain killers (g), feeding tube (h)

Abbreviations: AIC, Akaike Information Criterion; BIC, Bayesian Information Criterion; EORTC, European Organization for Research and Treatment of Cancer; LR, likelihood-ratio; MAE, mean absolute error; OLS, ordinary least-squares; QLQ-H&N35, Quality of Life Questionnaire-Head and Neck35; RMSE, root mean square error.

**Table 4:** Characteristics of the final model (Model 1d) without shrinkage.

EORTC QLQ-C30 scales	Coefficient	SE
Intercept	-1.0169	0.3721
Global health status/QoL	0.0210	0.0037
Physical functioning	0.0101	0.0038
Role functioning	0.0043	0.0027
Emotional functioning	0.0047	0.0026
Pain	-0.0126	0.0025

Abbreviations: EORTC, European Organization for Research and Treatment of Cancer; QLQ-C30, Quality of Life Questionnaire-Core 30; QoL, quality of life; SE, standard error.

### Predictive performance and validation of final model

**Fig 2:**
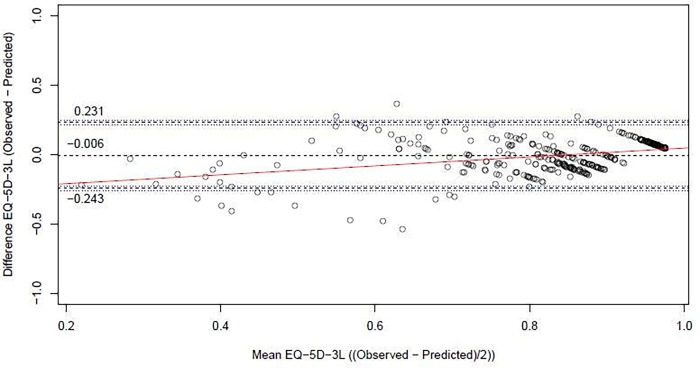
Bland-Altman plot of the final model with observed and predicted EQ-5D-3L values.

**Fig 3:**
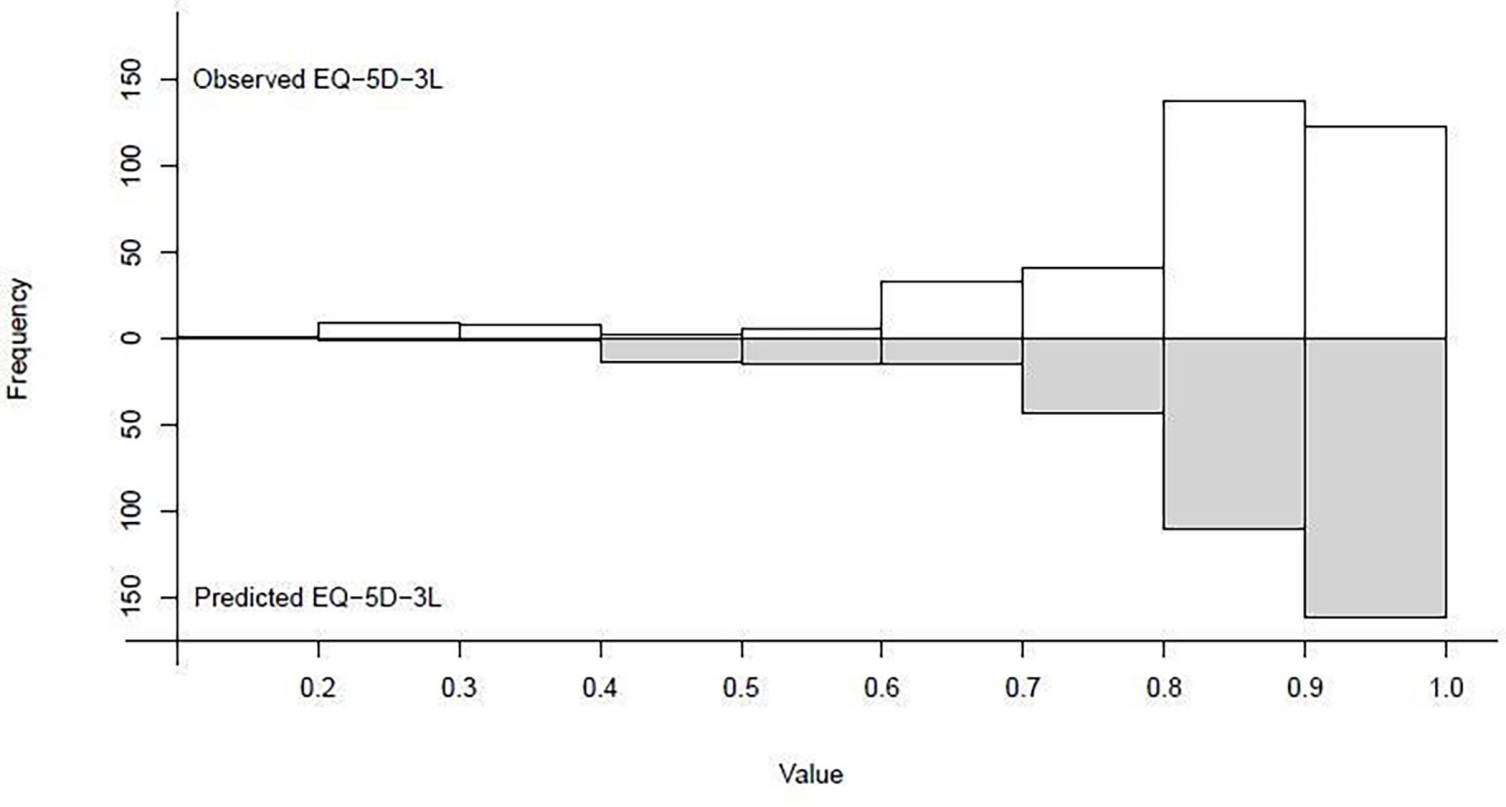
Mirrored histogram of observed and predicted EQ-5D-3L values.

The final model had an R^2^ of 0.3884. The MAE was 0.0949 and RMSE was 0.1209 (Table 3). The 95% limits of agreement were estimated at -0.243 to 0.231 (mean -0.006) (Fig 2). The Bland-Altman plot indicates that especially the utility values of patients with lower utility scores (mean <0.65) tend to be overestimated, reflected in a small positive error correlation of 0.32. Fig 3 shows histograms of the data for observed and predicted EQ-5D utility values, which also displays the overestimation in the lower values. The shrinkage factor of the coefficients using the heuristic formula was 0.90, indicating that minimal shrinkage of the coefficients is necessary for future predictions in new patients.

In assessing the strength of the predictive performance and internal validation of our model, the bootstrapping procedure of the fit indices indicated that the estimates of model fit were robust (bias MAE: 0.001; bias RMSE: 0.002).

## Discussion

In this study, we mapped the EORTC QLQ-C30 scales onto the EQ-5D-derived utility values using data from a Dutch cohort of 236 HNC patients. The beta regression model including the physical functioning, role functioning, emotional functioning, pain and global health status/QoL scales (Model 1d) was considered robust in terms of predictive performance. Taking into account dependency between repeated measurements by means of the multilevel approach (Model 1b) did not improve the model fit. The model had reasonable performance when comparing the MAE and RMSE to previously published models mapping the C30-scales onto the EQ-5D [4, 5, 7, 13, 30, 31]. Adding additional EORTC QLQ-C30 scales did not contribute to a better fit of the model. Contrary to our expectation, adding HNC-specific scales to the model led to inconsistent results in the model based on our criteria, whereas the improvements observed in MAE and/or RMSE of the models with EORTC QLQ-H&N35 scales were found negligible. Similar to our findings, Rogers et al., investigated the relationship between the EQ-5D domains and the domains of the University of Washington quality of life questionnaire (UW-QOL), and found that the generic domains of the UW-QOL (including pain, activity, recreation, mood and anxiety) showed strong correlations with the EQ-5D domains, whereas the HNC-specific domains did not [32, 33].

In literature, most studies mapping the EORTC QLQ-C30 to the EQ-5D used linear regression models [4, 5, 7, 8, 13, 30, 31]. In our study, the beta regression method showed the best fit compared to the other modeling approaches (step 1). This was also observed in the study of Kahn et al. in which the beta regression model outperformed the linear and Tobit models for lung cancer patients [34]. Although recent guidelines for mapping models also advise applying beta-based regression [6], few studies to date have employed the beta regression method [12, 34] and further research on the usefulness of this approach is desirable.

Although our results are largely consistent with those of previous studies using disease-specific QLQ scales [4, 5], our study provides no conclusive evidence on the value of adding disease-specific scales to mapping models in HNC patients. However, HNC-specific symptoms are very likely to have an important impact on HRQoL and we argue that they should therefore be of importance in calculating utility values to be used in cost-effectiveness analyses (CEAs). Therefore, development of a preference-based questionnaire that includes HNC-specific items may ultimately be a more efficient approach for generating disease-specific utilities for this population.

Designing and conducting prospective studies to collect PBM data for economic evaluation is time- and resource consuming. Applying a mapping model on readily available retrospective data can be advantageous, as it enables the conduct of CEAs even in the absence of utility data. However, the use of a mapping model inevitably introduces some uncertainty in the outcomes due to prediction error. In this study, the MAE was estimated at 0.0949. This seems acceptable, although it just exceeds the previously reported minimal important difference (MID) for EQ5D utilities of 0.08 [35]. There are no generally accepted cutoff values available to assess whether a model is suitable to apply in practice. The wide LOA in the Bland-Altman analysis indicates that our mapping model is less suitable for estimating utility levels of individual patients, but this is, of course, rarely done. However, the bias was close to 0 (mean -0.006), and the larger differences most often occurred in the lower and less common utility values. The QoL outcome for the HNC sample (Table 2) is rather good and—as this is a representative sample obtained from a national population database, the problem of the bias will be limited in clinical practice. Also, as the mean bias is very small, this model is likely well suited for use at a group level, as is usually the case in CEAs, and could therefore be a relevant tool to use to assess the cost-effectiveness in the clinic. As a cautionary note, the average overestimation of utility for patients with lower valued health states may impact the estimation of difference in utilities when comparing a group with high utility values to a group with low values, diluting the contrast.

Because predicting utilities will always be associated with uncertainty, mapping models should be seen as a second best solution, and we believe that ensuring availability of direct utilities should be promoted for all future research. As long as a HNC-specific PBM has not yet been developed, this should be done by using the generic EQ-5D-5L, in prospective studies.

To our knowledge, this is the first study to develop a mapping model, using the EORTC QLQ-C30 as well as EORTC QLQ-H&N35 scores as input data. Some limitations of this study need to be taken into consideration. Our sample size was limited due to the relatively low incidence of HNC and the amount of (complete) data available. The degree of variation of utility values below one was limited in our sample, which may have impacted precision of the estimates in low values (Fig 2). The clinical consequences of this may be limited, as such low values may not occur frequently among HNC patients. Subtypes of HNC included in this study were assumed to have similar patterns in QoL response to treatment and disease. Because of this, and with the objective to reduce the risk of overfitting, the prediction models did not include tumor diagnosis or stage. Finally, the model was tested on internal validity, but external validation in a new cohort of patients is needed to confirm the robustness of the model, before it can be used with confidence. Strengths of this study include: the robust approach used in developing the mapping model, including preselecting covariates based largely on theoretical considerations, comparison of four different statistical methods, and the exploration of the added value of disease-specific scales.

The fact that there is no gold standard for developing mapping models is reflected in the various methods and model fit statistics applied in literature. This makes it difficult to compare our study with other studies [10, 12, 13, 36]. Reaching international consensus on the preferred approach to modeling method(s) would enhance comparability across different mapping studies. Until such consensus has been reached, we would recommend adopting a similar strategy to modeling and reporting as applied in the current study, for future research.

## Conclusions

In this study, we were able to develop a model that maps the EORTC QLQ-C30 scales to the EQ-5D-derived utility values for patients with HNC. The added value of EORTC QLQ-H&N35 scales to the model remains ambiguous. The final model, using the beta regression method, includes five EORTC QLQ-C30 scales: global health status/QoL, physical functioning, role functioning, emotional functioning and pain. This model can be used cautiously to obtain utilities of HNC patients in situations where direct utilities are not available, to support economic evaluation and thus facilitate the implementation of innovative treatments and devices for HNC in clinical practice. Further research should assess the robustness and generalizability of the mapping model by validating the model in an external cohort of HNC patients.

## Abbreviations

AIC: Akaike Information Criterion
BIC: Bayesian Information Criterion
CEA: Cost-effectiveness analysis
DHNA: Dutch Head and Neck Audit
EQ-5D: EuroQol five-dimensional questionnaire
ES: Effect size
HNC: Head and neck cancer
HNSCC: Head and neck squamous cell carcinoma
HRQoL: Health-related quality of life
LOA: Limits of agreement
LR: Likelihood-ratio
MAE: Mean absolute error
MID: Minimal important difference
NKI: Netherlands Cancer Institute
OLS: Ordinary least squares
PBM: Preference-based measures
QALY: Quality-adjusted life years
EORTC QLQ-C30: European Organization for Research and Treatment of Cancer Quality of Life Questionnaire-Core 30
EORTC QLQ-H&N35: European Organization for Research and Treatment of Cancer Quality of Life Questionnaire-Head and neck cancer-35
Radboudumc: Radboud University Medical Center
RMSE: Root mean square error
